# Perioperative Pain Assessment and Medication Safety in Office-Based Dental Anesthesia: A Practical Review

**DOI:** 10.1007/s11916-026-01577-2

**Published:** 2026-11-03

**Authors:** Aidan McCarthy, Fred E. Shapiro, Richard D. Urman

**Affiliations:** 1 Tufts University, Boston, MA USA; 2 Massachusetts Eye and Ear Infirmary, Harvard Medical School, Boston, MA USA; 3 Department of Anesthesiology, The Ohio State University College of Medicine and Wexner Medical Center, N437 Doan Hall, 410 W. 10th Avenue, Columbus, OH 43210 USA

**Keywords:** Office-based anesthesia, Dental anesthesia, Pain assessment, Medication safety, Local anesthetics, Discharge readiness

## Abstract

**Purpose of Review:**

We examined perioperative pain assessment, medication documentation, toxicity preparedness, emergency medications and preparedness, and discharge readiness in office-based dental practices. By bringing these considerations together, the goal is to provide a practice overview of what providers should consider incorporating into their office-based dental and other similar anesthesia practices.

**Recent Findings:**

Office- based dental anesthesia consistently delivers care to children and patients who cannot tolerate hospital treatment. Effective perioperative pain management is critically important for safe patient care in the dental office. Age-appropriate pain assessment, local-anesthetic safety, emergency preparedness, and recovery and discharge criteria are important components of office-based dental anesthesia practices. Dental offices are an important setting for patients who require sedation or general anesthesia; A practical framework may help clinicians consistently integrate safeguard elements into daily office-based care.

## Introduction

Office-based anesthesia has become more popular and comprehensive for dental care. Young children, patients with special care needs, and individuals who cannot afford, drive, or be at an inconvenient for them dental center location may prefer a dental office for their procedure. A registry analysis of over 7000 pediatric dental office-based anesthesia (OBA) cases had reported no serious complications occurred, but a 3% rate of predischarge or post discharge adverse events [1]. The findings of that study suggest that although complications are rare, perioperative adverse events continue to occur, and as the number and complexity of patients and procedures continues to increase in the office setting, patient safety remains the central dogma to patient care. Appropriate pain assessments, analgesic management, emergency preparedness, and medication safety remain as some key pillars to sustain patient safety.

In all dental cases, pain remains an outcome of significant interest [2]. Prospective pediatric studies have documented clinically meaningful pain after dental treatment under general anesthesia. Moreover, systematic reviews have shown inconsistent assessment methods, and variable analgesic strategies and documentation [3, 4]. Furthermore, evidence for routine intraoperative local anesthesia remains variable. Trials report low postoperative pain, while others show little to no improvement in recovery and even evidence of complications such as soft-tissue biting [2–6].

Medication safety is a prominent concern for all clinicians. Local anesthetics are routinely used in dentistry and can produce rare but potentially catastrophic systemic toxicity. Key to safe office practice is early recognition, resuscitation, and lipid emulsion therapy for severe Local Anesthetic Systemic Toxicity (LAST). Office-based teams should have clear protocols and appropriate resources for LAST preparedness. Moreover, local anesthetic systemic toxicity requires prompt recongition and coordinated emergency response, including access to appropriate medications such as intravenous lipid emulsion therapy when indicated [7–9].

Standardization can be difficult because dental offices may operate under state dental board rules, physician anesthesiologists may also be subject to medical board requirements, and professional guidelines are not identical to enforceable regulations. Legal epidemiology and policy surveillance provide structural approaches to examining how laws and regulatory requirements are developed and implemented across jurisdictions [10, 11]. Permit requirements, facility inspections, and emergency preparedness expectations may differ from each dental office because of their individual jurisdiction and practice model [12, 13]. Provisions may name explicit resources, while other may broadly refer to resources such as “appropriate emergency drugs.” However, absence of a requirement in a written protocol or rule should not be interpreted as evidence that safety resources and protocol is absent from some dental offices.

This review focuses on perioperative pain assessment, analgesic management, local anesthetic safety, emergency medications, and discharge criteria in office-based dental anesthesia. These clinical considerations are interrelated, but each addresses a different aspect of patient safety. By bringing these considerations together, this provides a useful practical overview of what providers should consider incorporating into office-based dental anesthesia practice. In addition, this ​provides a useful practical overview of what providers should consider incorporating into office-based dental anesthesia practice (Table 1).

**Table 1:** Clinical considerations for pain assessment, monitoring, and medication safety in office-based dental anesthesia

Care phase	Clinical domain	What the office should incorporate or consider	Documentation / readiness check
PREOPERATIVE	Baseline pain and risk assessment	Document pain severity or behavior using an age-appropriate approach; review chronic pain, prior analgesics, relevant medications, allergies, and medical risk.	Record baseline score/observations, patient and caregiver history, and key risk factors.
PREOPERATIVE	Analgesic and prescribing plan	Anticipate procedural and recovery pain; select an individualized pain-management plan and confirm applicable prescribing rules and guidance.	Document planned medications, dosing instructions, and responsible clinician.
INTRAOPERATIVE	Physiologic monitoring	Monitor consciousness, oxygenation, ventilation, circulation, and clinical status as appropriate for the patient and anesthesia depth.	Maintain a time-oriented anesthesia and monitoring record; communicate changes.
INTRAOPERATIVE	Local-anesthetic safety	Coordinate selection, concentration, route, timing, and cumulative doses when local anesthetics are given or repeated by different providers.	Record each administration and reconcile cumulative local-anesthetic exposure.
THROUGHOUT CARE	Medication management and communication	Reconcile administered medications; communicate dosing, response, and relevant interactions across the dental and anesthesia teams.	Use a shared medication record and a clear handoff process.
THROUGHOUT CARE	Emergency preparedness and rescue	Maintain protocols, supplies, team roles, and escalation plans for anesthesia emergencies, LAST, and opioid-related respiratory depression/overdose.	Verify rescue equipment, emergency drugs and indicated antidotes/lipid emulsion; rehearse transfer and rescue processes.
POSTOPERATIVE	Recovery and physiologic readiness	Evaluate recovery of consciousness, ventilation, oxygenation, circulation, and protective functions; account for patient age and procedure.	Document recovery observations and use appropriate, consistent recovery criteria.
POSTOPERATIVE	Pain reassessment and response	Reassess pain when the patient can be meaningfully evaluated, including after analgesic treatment when indicated; observe nonverbal pediatric patients.	Record pain assessment, treatment response, and any need for further care.
DISCHARGE	Discharge decision and handoff	Consider pain and other symptoms alongside physiologic discharge criteria, readiness for home, and caregiver understanding.	Document discharge readiness, responsible escort/caregiver, and unresolved concerns.
DISCHARGE	Instructions and follow-up	Provide individualized written pain-management instructions, medication precautions, warning signs, and contact or escalation guidance.	Confirm patient/caregiver understanding and document follow-up instructions.

This is a proposed clinical organization of domains of interest in Office Based Dental Anesthesia not a validated checklist, an exhaustive standard of care, or a summary of what every jurisdiction requires. *LAST, * local anesthetic systemic toxicity

## Discussion

Office-based dental anesthesia requires attention to recovery criteria, emergency protocols and medications, patient discharge criteria, and postoperative prescribing [12, 13]. However structured pain assessment and documentation of analgesic effectiveness are also important office setting. Baseline pain assessment, reassessment after administration of an analgesic medication, or any confirmation that pain that has been addressed before discharge should be considered alongside physiologic recovery.

Prior work has described variability in dental-anesthesia regulations. Professional guidance emphasizes physiologic stability and emergency rescue pain management, assessment, and treatment should be considered alongside these priorities [12, 13].

Pain assessment can be incorporated into standardized anesthesia workflows rather than considered only in prescribing-related provisions. A preoperative or baseline pain assessment can help providers document a patient’s pain status and guide perioperative pain management and subsequent reassessment.

Pain scales are used throughout hospitals, surgery centers, and offices to help determine patients’ status and pain severity. Prior regulatory analysis found consistent variability in dental anesthesia regulations and guidelines, emphasizing the importance of pain scales and analgesic reassessment in, dentistry, especially when pediatric patients are more common [2, 12]. Pediatric patients may not be able to communicate pain as effectively and may require age-appropriate observational tools. Many clinical studies use tools such as the Faces Pain Scale–Revised, Parents’ Postoperative Pain Measure, and Faces-Legs-Activity-Cry-Consolability (FLACC) scale, and these assessments can complement physiologic or functional discharge readiness [2, 5].

Patients who are non-verbal or have special care needs may benefit from a preoperative caregiver interview so clinicians could intensify usual behaviors and baseline anxiety, anything that could correlate to signs of pain. During such interview, clinicians can ask caregivers about facial expressions, vocalization, guarding, agitations, or behaviors that are commonly indicative of discomfort. The information that is gained through a talk can be used to correlate behaviors in postoperative recovery. For children with cognitive impairment, the revised FLACC tool gives clinicians caregiver identified behaviors and has evidence of reliability and validity [14]. Incorporating caregiver observations into pain assessments may help improve pain management and recognition for patients who cannot reliably communicate their pain.

Discharge criteria commonly rely on vital signs, motor function, and airway stability (e.g., Aldrete scores). Although these criteria are important, an additional assessment of pain and the response to analgesic medication can support a more comprehensive evaluation before discharge.

Office-based dental anesthesia and surgery functions mainly off of pediatric patients. These patients may not be able to fully articulate pain effectively or verbally at all. This can create distress amongst the pediatric population, behavioral agitation, and soft tissue injury due to lip biting or cheek biting from local anesthesia [2, 5, 6].

Office-based dental anesthesia providers could transition from purely physiologic discharge criteria to a holistic discharge framework that incorporates validated observational pain tools, such as the Faces-Legs-Activity-Cry-Consolability scale or Faces Pain Scale–Revised (FPS-R), alongside vital signs before a patient is discharged home.

Furthermore, local-anesthetic documentation deserves similar attention. Documentation should address the drug name, concentration, dosage, timing, route, and total amount administered, while toxicity surveillance should also be considered. This may be clinically important when a dentist administers or repeats local anesthetic while an anesthesiologist manages sedation or general anesthesia, because incomplete communication may contribute to cumulative-dose errors, unrecognized drug interactions, or delayed recognition of toxicity. A unified, time-oriented medication record may support real-time communication between both clinicians and reduce medication-related adverse events [7–9, 13].

Office-based practices should consider a formal LAST protocol and appropriate access to intravenous lipid emulsion when local anesthetic is used in the office-based practice [8, 9]. LAST is seen to progress very rapidly from a neurogenic state to cardiovascular collapse, so broad categorical approaches to regulations, for example “appropriate emergency drugs” may not ensure immediate lipid emulsion access or a coordinated response [7–9].

Medication safety includes different but complementary processes, each addressing a significantly different aspect of patient safety. Prescription monitoring, electronic prescribing, or counseling requirements are different from, clinical decisions about postoperative pain treatment, medication reconciliation, or pain reassessment, and each should be evaluated. Independently. Preparedness to recognize and manage opioid-related respiratory depression or overdose is also an important part of emergency planning when such medications are used.

Contemporary dental practice to manage pain includes nonsteroidal anti-inflammatory drugs as first-line therapy for acute dental pain, and office-based surgery frameworks likewise emphasize multimodal strategies that minimize opioid exposure [15, 16]. Opioid-free approaches have also been described in pediatric dental day-case anesthesia [17]. These publications support nonopioid analgesia as an important clinical direction, but they do not replace the need for pain assessment, medication reconciliation, and rescue preparedness.

Corticosteroids, in many cases are used as perioperative adjuncts for procedures associated with postoperative inflammation. One example, dexamethasone, has been used clinically before and during third molar surgery, and was studied to reduce postoperative edema, trismus, and pain [18, 19]. When incorporated into a multimodal analgesic approach alongside NSAID’s and acetaminophen, dexamethasone may improve postoperative recovery. The effectiveness however can vary depending on the route of administration, dosage, and procedure performed. Its usage should be individualized based on patient specific factors and clinical contraindications. Improvements in postoperative swelling or pain should not be determined to automatically reduce opioid consumption.

These clinical considerations support consideration for a limited set of minimums, office-based practices rather than a prescribed analgesic regimen. Such expectations could include baseline pain assessment using an age-appropriate method, documentation of the planned analgesic strategy, reconciliation of all local-anesthetic agents and cumulative doses, recovery pain assessment, reassessment after rescue medication, written post discharge instructions, and a documented response pathway for local-anesthetic toxicity.

Dentists are major prescribers of immediate-release opioids for acute post-operative pain, such as wisdom tooth extractions, making dental care a primary point of initial opioid exposure for adolescents and young adults. Roughly 6% to 7% of opioid-naïve young adults transition to persistent use or receive an opioid abuse diagnosis within one year of receiving a dental prescription [20]. Similarly, persistent opioid use was present in 2.1% of patients who received an initial dental opioid prescription, compared with 1.0% of patients who did not [21]. However, routine dental opioid prescribing is often clinically unnecessary: patients managing post-extraction pain with opioids report significantly higher pain levels compared to those using non-opioid options, while maintaining comparable patient satisfaction [22]. A randomized trial found that ibuprofen-acetaminophen provided better early pain control and greater satisfaction than hydrocodone-acetaminophen following impacted third-molar extraction [23]. Beyond risk of dependence and overdose from diverted leftover medications, chronic opioid misuse has been associated with damages oral health, causing xerostomia, significant caries, and periodontal disease [24]. Despite recent reductions in prescribing rates, shifting to evidence-based non-opioid strategies—such as NSAID-acetaminophen combinations—remains essential to mitigating risks.

Office-based dental anesthesia should strongly emphasize physiologic safety, including maintenance of hemodynamic and physiologic stability, emergency preparedness, recovery, discharge readiness, and medication risk management. Standardized minimum expectations for pain documentation, local-anesthetic dose reconciliation, analgesic reassessment, and toxicity-response readiness may improve consistency while preserving clinical flexibility.

Prior studies have found regulations for each state across the United States place a strong priority on emergency preparedness and drugs, postoperative pain management, and discharge readiness [12]. In contrast, regulations draw short when having explicit perioperative pain assessments, pain documentation, local anesthetic safety, and LAST preparedness warrant focused attention. There is an opportunity to develop evidence-based clinical aids or checklists to help standardize recommendations for pain assessment and local anesthetic safety to promote consistency, while allowing clinicians to use their professional judgment and experience while practicing in the dental office.

**Fig. 1:**
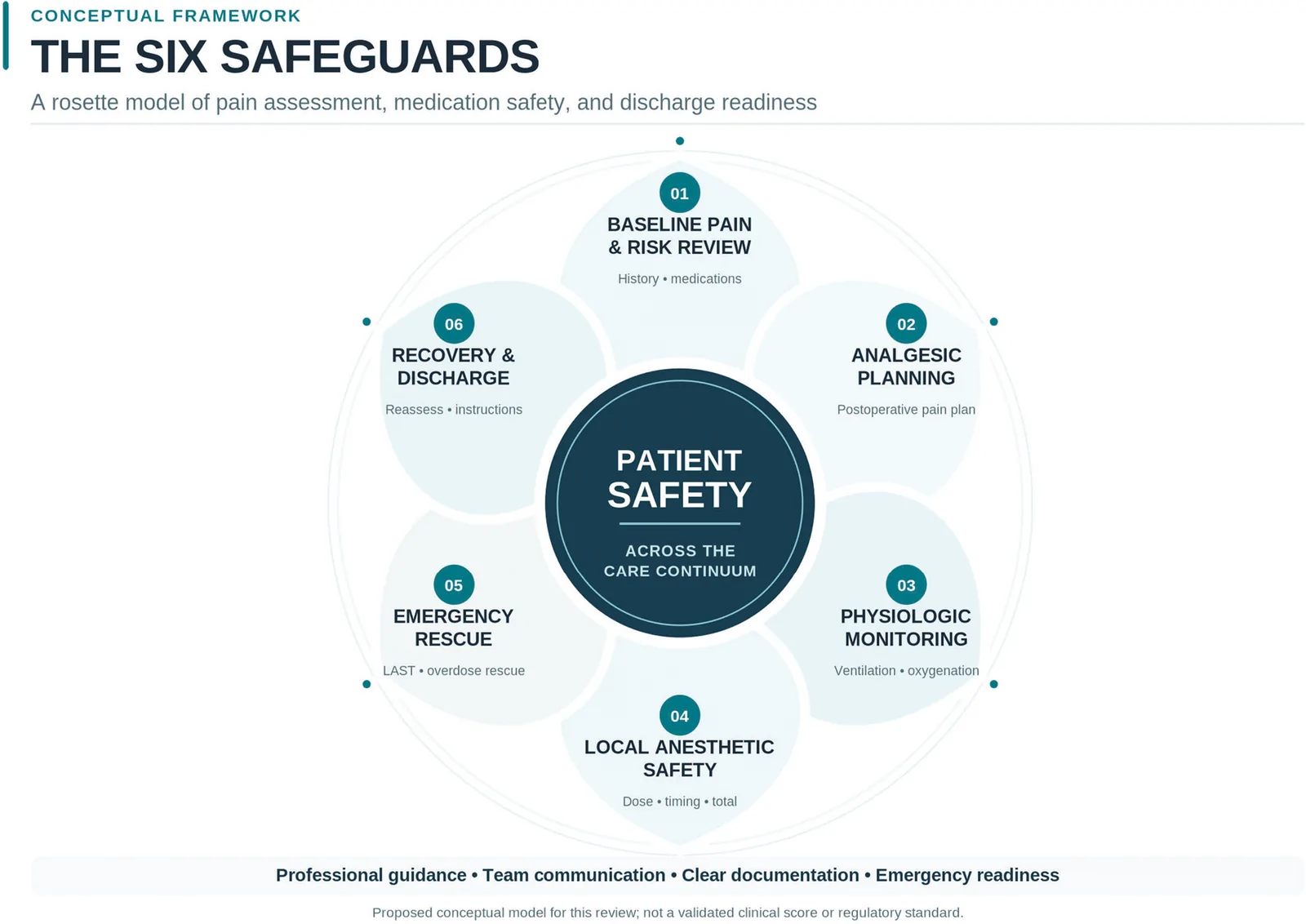
Six connected considerations in office-based dental anesthesia. The rosette emphasizes that preoperative pain and medication risk assessment, analgesic planning, physiologic monitoring, local anesthetic safety, emergency preparedness, and postoperative pain reassessment/discharge readiness collectively support patient safety. Clinical practices should follow applicable professional guidance and regulatory requirements. This conceptual illustration is not a validated instrument

These six safeguards may also be incorporated into the electronic health record (EHR) templates or paper anesthesia records (Fig. 1). Structural fields could prompt documentation of baseline pain scores, and caregiver reported behavioral cues, a planned analgesic strategy, and administration of local anesthetic agents with cumulative doses. Communication between both the dentist and anesthesia provider is critical, and using a time-oriented dose record could reduce any risk of cumulative dosage errors and facilitate early recognition of potential medication related complications. Recovery documentation can include postoperative pain reassessment, response to rescue medications if any, discharge readiness, and standardized written pain instructions. Checklists or prompts should support rather than replace clinical judgment and should be adapted to existing workflow and clinical standards.

This narrative review has several limitations. It is a focused clinical discussion rather than a systematic evidence synthesis, and it does not evaluate clinical practice patterns in individual dental. Offices. Professional guidance and office protocols may vary according to patient needs, the depth of anesthesia, and the practice setting. Thus, the considerations discussed here should be applied alongside current professional guidance, relevant regulatory requirements, and clinical judgment.

## Data Availability

All data supporting the findings of this study are available within the paper as Table​ and the supporting literature cited.

## Declarations

### Prior presentation

This work has not been previously presented.
